# Insights into protein degradation-related volatile substances changes in tilapia fillets (*Oreochromis niloticus*) during storage at 4 °C based on astral DIA proteomics and flavoromics

**DOI:** 10.1016/j.fochx.2026.103895

**Published:** 2026-04-21

**Authors:** Zhenyang Liu, Naiyong Xiao, Lei Qin, Zefu Wang, Qinxiu Sun, Hui Cao, Shucheng Liu

**Affiliations:** a College of Food Science and Technology, Guangdong Ocean University, Guangdong Provincial Key Laboratory of Aquatic Product Processing and Safety, Guangdong, Province Engineering Laboratory for Marine Biological Products, Guangdong Provincial Engineering Technology Research Center of Seafood, Key Laboratory of Advanced Processing of Aquatic Product of Guangdong Higher Education Institution, Zhanjiang 524088, China; b Universidade de Vigo, Nutrition and Bromatology Group, Department of Analytical Chemistry and Food Science, Faculty of Science, 32004 Ourense, Spain; c Instituto de Agroecoloxía e Alimentación (IAA), Universidade de Vigo, Campus Auga, 32004 Ourense, Spain; d School of Food Science and Technology, Dalian Polytechnic University, Collaborative Innovation Center of Seafood Deep Processing, Dalian Polytechnic University, Dalian 116034, China

**Keywords:** *Oreochromis niloticus*, Volatile substances, Astral DIA proteomics, Flavoromic, Formation pathways, Refrigerated storage

## Abstract

The aim of this study was to investigate the generation pathways of volatile substances derived from protein degradation in tilapia fillets stored at 4 °C, using Astral DIA proteomics. Seventeen key volatile substances were identified using HS-SPME-GC–MS. Among them, five substances were further recognized by the PLS-DA model as potential markers for sample classification. Differentially expressed proteins were primarily enriched in the Proteasome, Ribosome, and Oxidative phosphorylation pathways. Pearson correlation analysis and KEGG enrichment analysis suggested that major metabolic pathways, including Ribosome, Oxidative phosphorylation, and Neuroactive ligand-receptor interaction, may be associated with the formation of twelve key volatile substances, such as trimethylamine and nonanal, through protein degradation and the regulation of energy and lipid metabolism. A potential metabolic pathway map was constructed for amino acid, lipid, nucleotide, and carbohydrate metabolism. This study expands the theoretical understanding of volatile substances formation from protein degradation and provides insights into the regulation of protein degradation and flavor quality during refrigerated storage.

## Highlights

• Five potential classification marker volatile substances were identified by PLS-DA.
• Proteasome, ribosome and oxidative phosphorylation are the key degradation pathways.
• Proteasome, cytochrome *c* oxidase, etc. lead to apoptosis and protein degradation.
• Protein degradation, energy, and lipid metabolism affect production of key volatiles.
• Enzymes in metabolism of amino acid, lipid, etc. promote generation of key volatiles.

## Graphical abstract

**Figure:**
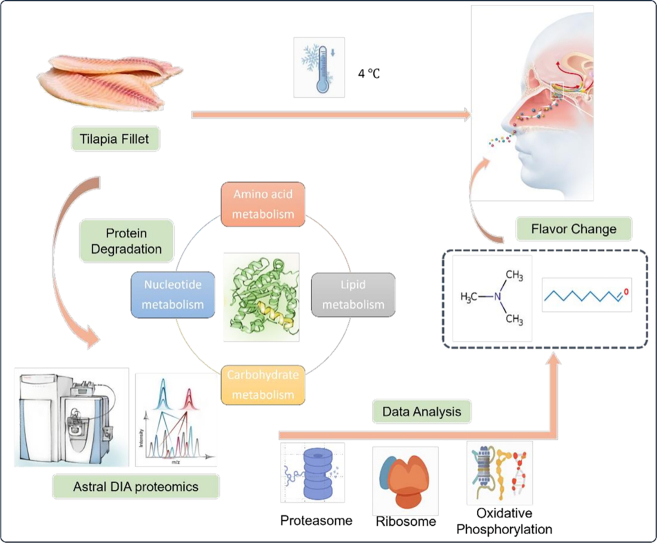

## 1 Introduction

In 2023, China's aquaculture production of tilapia (*Oreochromis niloticus*) reached 1.81 million tons, representing a 4.48% increase compared with 2022 (China & Bureau., 2024). In recent years, pre-processed tilapia products, particularly tilapia fillets, have gained increasing market acceptance owing to their convenience and ease of preparation. However, during the circulation, sale, and storage of tilapia fillets, large amounts of volatile substances, mainly off-flavor substances, are generated as a result of abundant endogenous enzymes and microbial proliferation. The deterioration of flavor markedly influences consumer purchasing behavior, as off-flavors are often perceived as indicators of spoilage and poor quality (Walayat et al., 2023). Therefore, this study aimed to elucidate the formation pathways of volatile substances from a protein degradation perspective.

Volatile substances are essential sensory attributes of aquatic products and serve as critical indicators for evaluating their quality. Current research has extensively characterized their types and concentrations in aquatic organisms. These studies have mainly focused on freshness assessment, off-flavor detection, evaluation of processing effects on flavor quality, and identification of specific aroma-active substances (Jiang et al., 2026; Liu et al., 2021; Walayat et al., 2023). The formation of volatile substances in aquatic products is closely associated with protein metabolism. Following harvest, these volatile substances mainly originate from precursor materials generated during protein degradation. Protein degradation is governed primarily by two mechanisms: (i) autolysis driven by endogenous enzymes and (ii) proteolysis mediated by microbial proteases. Proteins are initially hydrolyzed into small peptides and subsequently into free amino acids. These amino acids can be further converted into volatile compounds, such as aldehydes, alcohols, amines, and hydrocarbons, through deamination and decarboxylation reactions (Li, Geng, et al., 2022). Proteins also influence volatile substances through covalent and non-covalent interactions between specific amino acid residues and aroma-active molecules, thereby affecting their retention, perception, and release. Previous studies have explored the binding interactions of volatile substances such as furans (Yin et al., 2022) and pyrazines (Shen et al., 2019) with proteins. Protein structure can be altered by temperature, pH, pressure, oxidative stress, and processing methods. These changes may modify volatile-binding sites and consequently reshape volatile profiles (Shen et al., 2019). To date, research on volatile substances and proteins has predominantly focused on interaction mechanisms and protein conformational changes. However, studies elucidating the specific biochemical pathways of volatile substance formation driven by protein degradation remain scarce. The newly developed Astral data-independent acquisition (DIA) proteomics platform, based on the Orbitrap™ Astral™ high-resolution mass spectrometer, has substantially expanded the analytical capability of proteomics. By enabling high-speed, comprehensive, and unbiased acquisition of ion information, this technology provides improved detection depth, quantitative precision, and reproducibility compared with conventional proteomic approaches. In particular, its ability to detect low-abundance proteins and support robust multi-time-point comparisons makes it highly suitable for investigating dynamic postmortem changes during refrigerated storage. These features are particularly valuable for aquatic product flavor research, because they enable a more comprehensive characterization of storage-induced protein changes associated with proteolysis, energy metabolism, oxidative processes, and volatile substances formation. Astral DIA proteomics has been successfully applied to investigate color development (Tao et al., 2021), textural properties (Huang et al., 2024), and fermentation mechanisms (Planells-Cárcel et al., 2025), thereby identifying key proteins associated with quality changes at the molecular level. Nevertheless, its application in elucidating protein-driven pathways related to volatile substances formation remains limited. Therefore, its integration with flavoromics in the present study provides a novel strategy for linking protein-level changes with volatile evolution and for gaining deeper insight into the mechanisms underlying flavor deterioration in tilapia fillets.

Therefore, this study focuses on elucidating the formation pathways of volatile substances derived from protein degradation in tilapia fillets during storage at 4 °C, using an Astral DIA proteomics-based approach. Volatile substances and differentially expressed proteins (DEPs) were identified and analyzed through HS-SPME-GC–MS and Astral DIA proteomics, respectively. The metabolic pathways responsible for the generation of key volatile substances from protein degradation were further explored via Pearson correlation analysis and Kyoto Encyclopedia of Genes and Genomes (KEGG) enrichment analysis. Overall, this study aims to clarify the mechanistic relationship between protein degradation and volatile substances formation. It also seeks to provide theoretical insights for regulating protein degradation and improving flavor quality during refrigerated storage.

## 2 Materials and methods

### 2.1 Sample preparation

Fresh tilapia (*Oreochromis niloticus*, approximately 1.25 kg per fish) were purchased from Huguang Market (Zhanjiang, Guangdong, China), and 10 fish were used in this study. The fish were slaughtered by market staff prior to purchase. The dorsal muscles were collected immediately after purchase, transported to the laboratory in an ice box within 2 h, rinsed thoroughly, and sliced into fillets of uniform thickness (3.5 mm) using an electric multifunctional slicer (QRLS-400II, Zhenjiang Dagang Guangming Machinery Factory, China). The fillets were evenly arranged in bags, vacuum-packaged using a vacuum packaging machine (FKJ-G01F2, Guangdong Meihao Electric Co., China), and stored at 4 °C to simulate retail conditions. Filleting and vacuum packaging were conducted immediately under chilled conditions to minimize biochemical changes before storage. Samples were collected on days 0, 1, 3, 5, and 7, and all samples were stored at −80 °C until analysis.

### 2.2 HS-SPME-GC–MS analysis of volatile substances

Fish fillets were collected at different times. 5 g of fish meat, 5 mL of saturated sodium chloride solution (Saturated sodium chloride solution was added to the headspace vial to induce a salting-out effect, which facilitates the release of volatile substances into the headspace and improves the extraction efficiency of HS-SPME.), and internal standard solution (Nonanoic acid methyl ester, 25 mg/L in methanol; Anpu Experimental Technology Co., Shanghai, China) were added to a headspace vial. The vial was placed in a magnetic stirring bath (LC-OB-5 L, Li-Chen Instrument Technology Co., Shanghai, China) at a constant temperature of 60 °C for 5 min. Subsequently, 50/30 μm Divinylbenzene/Carboxen/polydimethylsiloxane (DVB/CAR/PDMS) fiber was inserted into the vial for headspace adsorption for 35 min. Desorption was performed by inserting the fiber into the injector of the GC–MS-TQ8040NX instrument (Shimadzu Co., Ltd., Japan) at 250 °C for 5 min. Volatile substances were separated in a fused silica capillary column (SH-Rtx-Wax，60 m × 0.25 mm × 0.25 μm, Quadrex, Woodbridge, CT, USA). Helium (purity of 99.999%) was used as the carrier gas at a flow rate of 1.0 mL/min in splitless mode. The temperature for the Chromatographic column was set as follows: initial temperature of 40 °C held for 1 min, increased at 4 °C/min to 200 °C (held for 2 min), then ramped up to 230 °C at 5 °C/min. The ionization energy and ion source temperature were set at 70 eV and 230 °C, respectively. The qualitative and quantitative analyses of volatile substances, along with the calculation of odor activity values, were performed according to a previously published method (Liu et al., 2021). The volatile substances detected by GC–MS were identified by comparing their mass spectra with those in the NIST 2.0 and Wiley 6.0 mass spectral libraries installed on the GC–MS system. Identification was considered reliable when the similarity score exceeded 80 and the retention index (RI) matched the reference value. A semi-quantitative approach was used to estimate the concentrations of volatile substances by relating the peak areas of the analytes to that of the internal standard (IS). Based on eqs. (1), (2), and (3), the odorant concentration, odor activity value (OAV), and RI values were calculated.

(1) $$\mathrm{Wi}=f^{'}\times \left(\mathrm{Ai}\times \mathrm{Ws}\right)/\mathrm{As}$$

(2) OAV=Wi/Ci

(3) $$\mathrm{RI}=100\times n+100\times \left[\text{logtR}\left(x\right)–\text{logtR}\left(n\right)\right]/[\text{logtR}\left(n+1\right)-\text{logtR}\left(n\right)$$

The symbols of Wi, f', Ai, Ws, As, and Ci are the odorant concentration (μg/g), calibration factors (assumed as 1.0), the peak area of the compound, the IS concentration (μg/g), the IS peak area, and the sensory threshold of an odor in water, respectively. R(x) was the adjusted retention time of the test compound x, min. R(n) was the n-alkane with a carbon number of n, and the retention time was min, and R (n + 1) was a normal structure with n + 1 carbon atoms. The alkane was used to adjust the retention time, and n means the number of carbon atoms.

### 2.3 Ethics statement

The fish used in this study had been slaughtered by market staff prior to purchase. Specifically, the fish were stunned by a blow to the head with a wooden stick, and the viscera, head, and tail were then immediately removed. The dorsal muscles were collected immediately after purchase. All animal-related procedures complied with the guidelines of the Experimental Animal Ethics Committee of Guangdong Ocean University (Approval No. GDOU-LAE-2023-043).

### 2.4 Protein extraction and digestion

Samples were first homogenized by MP FastPrep-24 homogenizer (24 × 2, 6.0 M/S, 60 s, twice)，and then SDT buffer (4% SDS, 100 mM Tris-HCl, pH 7.6) was added. DTT (with the final concentration of 40 mM) was added to each sample respectively and mixed at 600 rpm for 1.5 h (37 °C). After the samples had cooled to room temperature, IAA was added to a final concentration of 20 mM to block reduced cysteine residues. The mixtures were then incubated in the dark for 30 min. Next, the samples were transferred to the filters (Microcon units, 10 kDa) respectively. The filters were washed with 100 μL UA buffer three times and then 100 μL 25 mM NH_4_HCO_3_ buffer twice. Finally, trypsin was added to the samples (the trypsin: protein (wt/wt) ratio was 1:50) and incubated at 37 °C for 15–18 h (overnight), and the resulting peptides were collected as a filtrate. The peptides of each sample were desalted on C18 Cartridges (Empore™ SPE Cartridges MCX, 30UM, waters), concentrated by vacuum centrifugation and reconstituted in 40 μL of 0.1% (*v*/v) formic acid. The peptide content was estimated by UV light spectral density at 280 nm. For DIA experiments, iRT (indexed retention time) calibration peptides were spiked into the sample.

### 2.5 Mass spectrometry assay for data independent acquisition (DIA)

The peptides from each sample were analyzed by Orbitrap™ Astral™ mass spectrometer (Thermo Fisher Scientific Inc., Waltham, USA) connected to an Vanquish Neo system liquid chromatography (Thermo Fisher Scientific Inc., Waltham, USA) in the data-independent acquisition (DIA) mode. Precursor ions were scanned at a mass range of 380–980 *m*/*z*，MS1 resolution was 240,000 at 200 m/z，Normalized AGC Target: 500%，Maximum IT: 5 ms. 299 windows were set for DIA mode in MS2 scanning, Isolation Window: 2 m/z，HCD Collision Energy: 25 ev, Normalized AGC Target: 500%，Maximum IT: 3 ms.

### 2.6 Protein mass spectrometry data analysis and bioinformatics analysis

DIA data was analyzed with DIA-NN (Version 1.8.1). Main software parameters were set as follows: enzyme is trypsin, max missed cleavages is 1, fixed modification is carbamidomethyl (C), dynamic modification is oxidation (M) and acetyl (Protein N-term). All reported data were based on 99% confidence for protein identification as determined by false discovery rate (FDR) ≤ 1%.

DEPs across different groups were screened using fold-change and significance thresholds. Proteins with a fold change >1.5 or < 0.67 and a *P* value <0.05 were considered differentially expressed. By utilizing the Blast2GO software (http://www.geneontology.org/) for the annotation of DEPs based on Gene Ontology (GO) terms, the annotated proteins were further mapped to the Kyoto Encyclopedia of Genes and Genomes (KEGG) pathway database (http://geneonto logy.org/) for their localization on specific KEGG pathways. Enrichment analysis was applied based on the Fisher’ exact test.

### 2.7 Statistical analysis

All experiments were conducted in triplicate, and the results were averaged. One-way analysis of variance (ANOVA) was performed using JMP software (SAS, North Carolina, USA) to determine significant differences among groups. Significance was considered when the *P*-value was less than 0.05. Principal component analysis (PCA) and partial least squares-discriminant analysis (PLS—DA) of volatile substances were performed using MetaboAnalyst.

## 3 Results and discussion

### 3.1 Changes of key volatile substances in Tilapia fillets during storage

During refrigerated storage, 43 volatile substances were detected in tilapia fillets (Table S1), including alcohols (7), ketones (4), aldehydes (11), acids (8), esters (2), nitrogen-containing compounds (1), sulfur-containing compounds (1), and hydrocarbons (9). The aroma of the fillets reflects both the abundance of these substances and their sensory potency, which is governed by odor thresholds. Accordingly, odor activity values (OAVs)-derived from measured concentrations relative to threshold values-were used to screen aroma-active volatile substances, with OAV > 1 indicating major contributors to the characteristic fish flavor (Jiang et al., 2026). The OAV is defined as the ratio of the actual concentration of a volatile substance in a food matrix to its corresponding odor threshold. The odor threshold represents the minimum concentration at which the volatile substances can just be perceived by the human nose. After entering the nasal cavity, volatile molecules interact with receptors in the olfactory epithelium. A substance can only be perceived as an odor when its effective partial pressure in the gas phase, transfer efficiency, and the resulting receptor stimulation reachs a sufficient level. Therefore, when the OAV is equal to or greater than 1, the concentration of the volatile substances in the given matrix has reached or exceeded the sensory perception threshold and the signal level required to rise above the background noise, indicating that it theoretically has the potential to be recognized by the olfactory system.

Seventeen volatile substances showed OAVs >1 during storage (Table S2) and were therefore identified as key volatile substances. Among them, aldehydes were the predominant group, including pentanal, hexanal, heptanal, octanal, nonanal, decanal, (*2E*)-2-decenal, (*E*)-2-nonenal, and benzaldehyde. Except for benzaldehyde, most aldehydes increased progressively during storage. Given their low odor thresholds, these aldehydes were the major contributors to the aroma characteristics and off-flavor development of tilapia fillets. Aldehydes are typically formed through lipid oxidation, protein degradation, amino acid deamination, and decarboxylation processes in fish muscle tissue (Li, Liu, et al., 2022). Alcohols constituted the second major group of key volatile substances, including hexanol, 1-octen-3-ol, heptanol, and octanol. Most of them also increased during storage, except for heptanol. Alcohols are formed mainly through lipid oxidation, microbial metabolism, and amino acid degradation. Generally, linear-chain alcohols are derived from lipid oxidation, while branched-chain alcohols are produced via microbial degradation of branched aldehydes (Shi et al., 2019). However, owing to their relatively high odor thresholds, alcohols contribute less to the overall flavor of tilapia fillets compared with aldehydes. In addition, trimethylamine, 6-methyl-5-hepten-2-one, butyric acid, and eugenol were identified as key volatile substances. Among them, trimethylamine increased markedly during storage and is considered an important spoilage-related substances responsible for fishy odor (Walayat et al., 2023). By contrast, butyric acid and eugenol showed decreasing trends. Ketones and phenolic substances were also detected. However, their contribution to flavor was limited due to either high odor thresholds (ketones) or low concentrations (phenols) (Zhang, Zhou, et al., 2019).

Overall, pentanal, hexanal, heptanal, octanal, nonanal, decanal, (*E*)-2-nonenal, 1-octen-3-ol, and trimethylamine made dominant contributions to the total OAV of tilapia fillets. This was mainly due to their relatively high concentrations and low odor thresholds. These substances mainly imparted fishy, fatty, mushroom-like, and earthy notes and were therefore considered the major drivers of off-flavor formation in tilapia fillets, consistent with previous reports (Zhou et al., 2023). In contrast, substances such as 6-methyl-5-hepten-2-one, hexanol, octanol, benzaldehyde, (*2E*)-2-decenal, butyric acid, and eugenol contributed fruity, grassy, fatty, and buttery, suggesting that some volatile substances formed during storage may also provide limited positive aroma attributes (Wu et al., 2024).

### 3.2 Multivariate statistical analysis of volatile substances

**Fig. 1:**
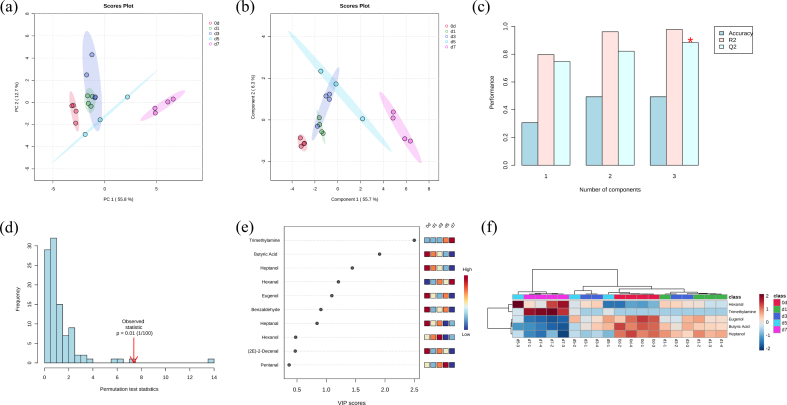
Multivariate statistical analysis of volatile substances in tilapia fillets during refrigerated storage. (a) Principal component analysis (PCA) score plot, (b) Partial least squares–discriminant analysis (PLS-DA) score plot, (c) Cross-validation results of PLS-DA model, (d) Permutation test of the PLS-DA model, (e) Variable importance in projection (VIP) scores of volatile substances, (f) Hierarchical cluster analysis of key volatile substances (VIP > 1 and OAV > 1).

Principal component analysis (PCA) is a widely used multivariate statistical technique that reduces the dimensionality of complex flavor substance datasets through eigenvector transformation, enabling visualization of flavor differences among samples (Panebianco et al., 2022; Zuo et al., 2025). As shown in the PCA score plot (Fig. 1a), the first principal component (PC1) and the second principal component (PC2) explained 55.8% and 12.7% of the total variance, respectively. The cumulative variance explained by these two components reached 68.5%, indicating that the PCA model effectively captured the major variation present in the dataset. The d0 and d7 samples were clearly separated from the other groups without overlap, indicating marked differences in volatile substances compared with the remaining samples. In contrast, the d1, d3, and d5 samples were located closer together with partial overlap, suggesting relatively minor differences in volatile substances composition among these groups. Partial least squares-discriminant analysis (PLS—DA) is a supervised classification approach that combines the strengths of PCA, canonical correlation analysis, and multiple linear regression analysis. This method efficiently extracts predictive information from high-dimensional datasets (Cheng et al., 2023; Feng et al., 2023). It also facilitates sample classification and helps identify substances that contribute most to group discrimination. As shown in Fig. 1c and d, the PLS-DA model exhibited strong predictive ability, with Q^2^ and R^2^ values of 0.884 and 0.978, respectively. The permutation test further demonstrated that the original model performed significantly better than randomly generated models (*P* = 0.01), indicating that overfitting was not observed. As illustrated in Fig. 1b, the d0 and d7 samples were clearly separated from the remaining groups without overlap, suggesting pronounced differences in volatile substances composition. In contrast, the d1, d3, and d5 samples were positioned closer together and showed partial overlap, indicating similarities in volatile profiles. This trend was consistent with the PCA results. To clarify changes in key volatile substances during refrigerated storage of tilapia fillets, variables with high contributions were screened using variable importance in projection (VIP) values derived from the PLS-DA model. In general, substances with VIP values greater than 1 are considered to contribute substantially to group discrimination. As shown in Fig. 1e, five volatile substances exhibited VIP values above 1, namely trimethylamine, butyric acid, heptanol, hexanal, and eugenol. These substances represent major differential volatile substances and may serve as potential markers for distinguishing storage stages of tilapia fillets (Cheng et al., 2023). Hierarchical clustering analysis was further visualized using a heatmap, as shown in Fig. 1f. This analysis was used to examine the relationships among key volatile substances with VIP > 1 and OAV > 1 during refrigerated storage. In the heatmap, red and blue colors indicate relatively high and low abundances, respectively. The d0 and d7 samples were distributed separately from the other groups. Higher levels of heptanol, butyric acid, and eugenol were observed in the d0 samples, whereas trimethylamine and hexanal were more abundant in the d7 samples. The d1 and d3 groups showed comparable contents of heptanol, butyric acid, and eugenol. In addition, butyric acid and eugenol presented similar levels among the d1, d3, and d5 samples, suggesting a certain similarity in volatile substances composition among these groups. These observations were generally consistent with the patterns obtained from PCA (Fig. 1a) and PLS-DA (Fig. 1b). These results indicate that storage time markedly influences the formation of volatile substances in tilapia fillets. In the d0 samples, heptanol, butyric acid, and eugenol contributed chemical, green, buttery, cheesy, sour, clove-like, smoky, and spicy odor notes. With prolonged refrigerated storage, the levels of these aroma substances gradually decreased in the d1, d3, and d5 samples. Trimethylamine is associated with rotten fish and ammonia-like odors, while hexanal contributes fishy, fatty, and green notes. The higher abundance of these substances in d7 samples suggests a decline in freshness of tilapia fillets at this stage.

### 3.3 Changes in proteins in Tilapia fillets during refrigerated storage

**Fig. 2:**
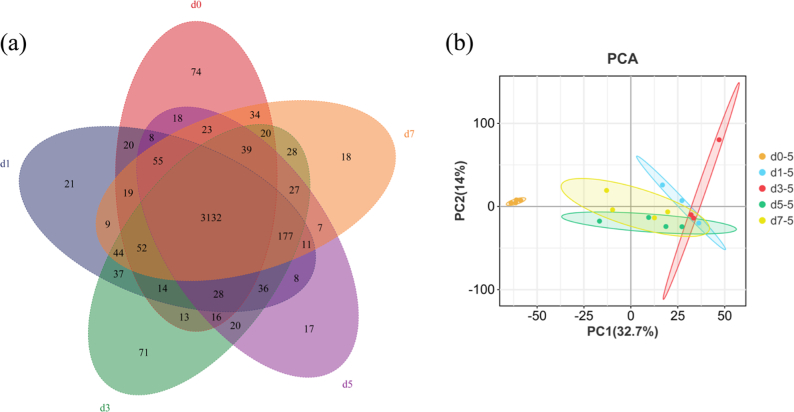
Analysis of protein in tilapia fillets during refrigerated storage. (a) Veen diagram, (b) PCA scores plot.

To investigate protein expression patterns during refrigerated storage and elucidate alterations in the proteome, Astral DIA proteomics analysis was performed on tilapia fillets stored for different durations. As shown in Fig. 2a, a total of 3132 proteins were identified across all sample groups. During storage, distinct changes in the proteome were observed, with 74, 21, 71, 17, and 18 unique proteins detected in the 0 d, 1 d, 3 d, 5 d, and 7 d groups, respectively. Principal component analysis (PCA) was used to evaluate inter-group variation based on DEPs. As shown in Fig. 2b, the d0 samples were clearly separated from all other storage groups, whereas the d1, d3, d5, and d7 samples clustered closely together. These results suggest that substantial proteomic alterations occurred immediately after the onset of storage, while further changes from d1 to d7 were comparatively limited (Liu et al., 2022). Notably, the separation between the d0 and d7 groups further supports the existence of distinct molecular characteristics between the early and late stages of refrigerated storage, which is also consistent with the marked evolution of volatile profiles observed during storage. Together, these findings suggest that refrigerated storage induced progressive biochemical changes in tilapia fillets at both the proteomic and flavor levels.

**Fig. 3:**
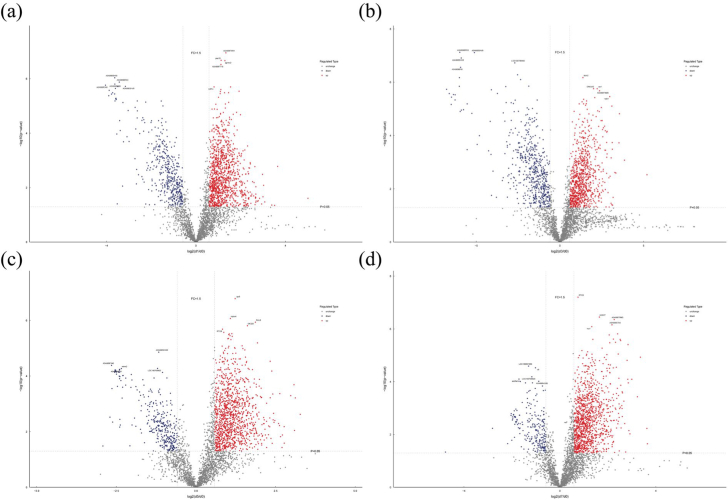
Volcano map of DEPs in tilapia fillets during refrigerated storage. (a) d1/d0, (b) d3/d0, (c) d5/d0, (d) d7/d0.

To further assess the effects of refrigerated storage on protein expression, DEPs were visualized using volcano plots based on fold-change and *P*-value criteria. Proteins with a fold change >1.5 and *P* < 0.05 were classified as significantly upregulated (red), whereas those with a fold change <0.67 and *P* < 0.05 were classified as significantly downregulated (blue). As shown in Fig. 3, Fig. 1,156, 911, 1192, and 1188 proteins were upregulated in the d1/d0, d3/d0, d5/d0, and d7/d0 comparisons, respectively, while 451, 564, 330, and 230 proteins were downregulated. The large number of DEPs identified across all comparisons clearly demonstrates that refrigerated storage profoundly affects the proteomic profile of tilapia fillets (Du et al., 2024).

### 3.4 Subcellular localization and domain analysis of DEPs

Proteins are fundamental cellular components that perform diverse biological functions. Specific proteins are localized in different organelles and execute distinct roles. Polypeptides composed of tens to hundreds of amino acids fold into characteristic three-dimensional structures (domains), which determine their biological activity. Interactions among proteins, or between proteins and small molecules, may induce structural modifications within protein domains. These changes may alter their functional properties. Therefore, subcellular localization and domain prediction analyses of DEPs are essential for elucidating how refrigerated storage affects protein function in tilapia fillets. As shown in Fig. S1, DEPs identified in each comparison group were annotated using a database to determine their subcellular distribution and functional domain characteristics. The DEPs were located in eight subcellular compartments, including the nucleus, lysosome, extracellular space, plasma membrane, mitochondria, cytoskeleton, cytoplasm, and peroxisome. Across all comparisons, DEPs were predominantly localized in the nucleus, cytoplasm, extracellular region, and mitochondria, together accounting for more than 90% of all identified DEPs. This finding indicates that proteins in these four cellular compartments are particularly susceptible to expression changes during refrigerated storage. Specifically, cytoplasmic proteins are closely associated with postmortem glycolysis, amino acid turnover, and proteolytic processes, which are major metabolic events occurring in fish muscle after slaughter (Christine, Romuald, Richard, & Véronique, 2006; Xiong et al., 2024). Mitochondrial proteins are likely related to residual energy metabolism and oxidative processes, as mitochondria remain important sites of reactive oxygen species generation and redox imbalance during postmortem storage. Such oxidative changes may accelerate lipid oxidation, protein oxidation, and the deterioration of overall muscle quality (Li, Liu, et al., 2022). In addition, the enrichment of extracellular region proteins may reflect disruption of cell membrane integrity and the release of matrix-associated or structural proteins during storage, which is closely linked to muscle softening and texture deterioration (Yao & Xie, 2026). Nuclear-localized proteins may be associated with the degradation, redistribution, or altered regulation of intracellular components after slaughter, reflecting progressive loss of cellular homeostasis in postmortem tissue.

Domain enrichment analysis revealed that DEPs were mainly associated with the Immunoglobulin I-set domain, RNA recognition motif, and Fibronectin type III domain. The presence of these domains suggests that proteins involved in immune regulation, RNA processing, and extracellular matrix interactions are significantly affected during storage. Such alterations may reflect the activation of defense-related responses in fish muscle, potentially to counter microbial challenge or antigen invasion. Moreover, during low-temperature storage, the activity of endogenous enzymes and microorganisms may impair membrane integrity and organelle stability, which could contribute to changes in muscle texture. Taken together, these results suggest that refrigerated storage induces substantial proteomic changes in tilapia fillets. These changes are mainly associated with pathways related to metabolic regulation, immune response, and energy metabolism (Du et al., 2024).

### 3.5 Analysis of the pathways from Tilapia protein degradation

**Fig. 4:**
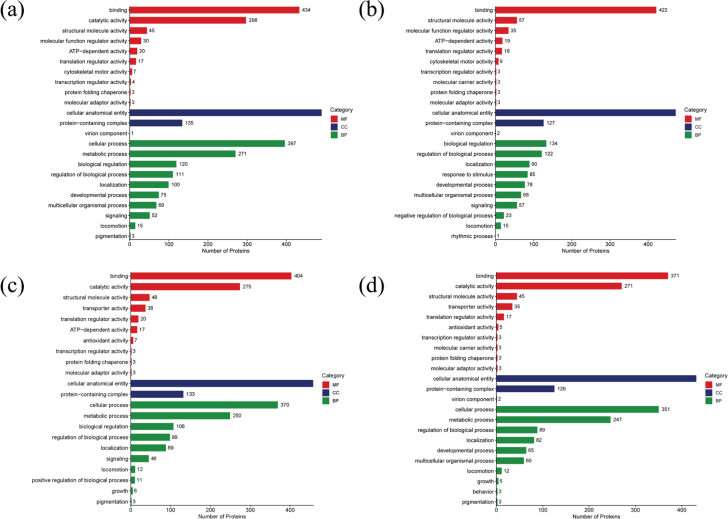
GO enrichment analysis of the DEPs in tilapia fillets during refrigerated storage. (a) d1/d0, (b) d3/d0, (c) d5/d0, (d) d7/d0.

To comprehensively elucidate the functions, localization, and biological pathways associated with proteins in organisms, Gene Ontology (GO) annotation was performed. GO terms are classified into three major categories: biological process (BP), molecular function (MF), and cellular component (CC). GO functional annotation of the DEPs identified in different comparison groups is presented in Fig. 4. Overall, the GO term distribution of DEPs was similar across all comparison groups, except for the d3 vs d0 group. In the MF category, DEPs in the d1 vs d0, d5 vs d0, and d7 vs d0 groups were primarily enriched in binding (434, 404, and 371 proteins, respectively, GO:0005488) and catalytic activity (298, 275, and 271 proteins, respectively, GO:0003824). Previous studies have reported that binding-related proteins are associated with quality traits such as color and pH, whereas catalytic activity-related proteins participate in protein degradation and energy metabolism (Pan et al., 2022). In the CC category, DEPs were mainly localized in cellular anatomical entities (491, 459, and 432 proteins, GO:0110165) and protein-containing complexes (135, 133, and 126 proteins, GO:0032991). In the BP category, DEPs predominantly participated in cellular processes (397, 370, and 351 proteins, GO:0009987) and metabolic processes (271, 250, and 247 proteins, GO:0008152), suggesting that refrigerated storage affects proteins involved in physiological regulation and metabolic pathways. Interestingly, the number of DEPs associated with MF, CC, and BP exhibited a decreasing trend during refrigerated storage. This phenomenon may be attributed to the stress response induced by low-temperature conditions, which could suppress physiological metabolism and reduce protein synthesis in fish muscle tissue (Zhang et al., 2023).

**Fig. 5:**
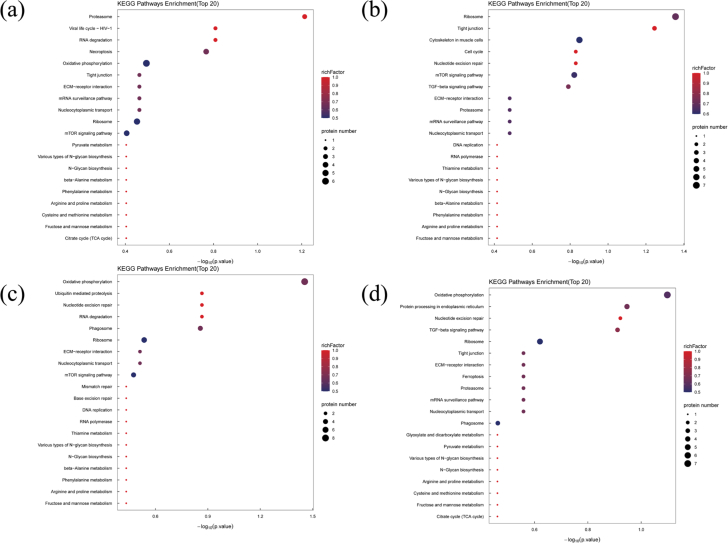
KEGG enrichment analysis of the DEPs in tilapia fillets during refrigerated storage. (a) d1/d0, (b) d3/d0, (c) d5/d0, (d) d7/d0.

To further elucidate the effects of refrigerated storage on metabolic pathways associated with DEPs in tilapia fillets, KEGG pathway enrichment analysis was conducted at different storage times. This analysis enabled the identification of biological functions, regulatory networks, and specific metabolic pathways affected by refrigerated storage. As shown in Fig. 5, the top 20 enriched KEGG pathways are presented for each comparison group, with 67, 66, 60, and 63 pathways identified in the d1/d0, d3/d0, d5/d0, and d7/d0 groups, respectively. Among these pathways, the Proteasome (onl03050), Ribosome (onl03010), and Oxidative phosphorylation (onl00190) pathways were significantly enriched during storage, as further illustrated in Fig. S2. In the Proteasome pathway (onl03050), DEPs included proteasome subunit beta (A0A669BB28, downregulated), proteasome 26S subunit ATPase 1 (I3IXY1, upregulated), and 26S proteasome regulatory subunit 9 (I3K6A9, upregulated). During refrigerated storage, upregulation of the 26S proteasome ATPase 1 and regulatory subunit 9 may accelerate the degradation of damaged or misfolded proteins, contributing to texture softening in fish muscle. Conversely, the downregulation of the proteasome beta subunit suggests that low temperatures may partially inhibit proteasomal activity, thereby reducing excessive myofibrillar breakdown and delaying structural deterioration (Yan et al., 2023). In the Ribosome pathway (onl03010), DEPs included large ribosomal subunit protein eL30 (I3J3A6, downregulated), 40S ribosomal protein S25 (A0A669E4L2, upregulated), ribosomal protein P1 (I3J3T8, upregulated), 60S ribosomal proteins L13 (I3JF29, upregulated) and L26 (I3KME3, downregulated), small subunit protein eS12 (O13019, upregulated), and large subunit protein eL18 (P69091, upregulated). Ribosomal proteins not only participate in protein synthesis but also contribute to DNA repair, cellular development, and stress regulation. Abnormal expression of ribosomal proteins has been associated with apoptosis, cell cycle arrest, protein denaturation, and changes in meat color and tRNA stability (Pan et al., 2022; Yan et al., 2023). The upregulation of ribosomal proteins observed here suggests enhanced protein turnover and increased cellular apoptosis during storage. In the Oxidative phosphorylation pathway (onl00190), all identified DEPs-including V-type proton ATPase subunit G (A0A669CF55, upregulated), NADH dehydrogenase [ubiquinone] iron‑sulfur protein 4, mitochondrial (A0A669CQS1, upregulated), Cytochrome c oxidase subunit (A0A669CXL8, upregulated), NADH dehydrogenase [ubiquinone] 1 alpha subcomplex subunit 6 (A0A669EMT2, upregulated), ATP synthase subunit d, mitochondrial (I3J116, upregulated), ATP synthase subunit O, mitochondrial (I3K590, upregulated), NADH dehydrogenase [ubiquinone] iron‑sulfur protein 8, mitochondrial (I3KGI6, upregulated), and Cytochrome c oxidase subunit (I3KVG2, upregulated)-were upregulated. Following slaughter, the cessation of nutrient supply and hypoxic conditions within fish muscle cells shift metabolism toward pathways that sustain ATP production. Oxidative phosphorylation becomes a primary energy source, utilizing amino acids, proteins, and sugars to maintain essential metabolic functions (Yan et al., 2023). Upregulation of ATP synthase, cytochrome *c* oxidase, and NADH dehydrogenase indicates intensified mitochondrial activity during storage. Furthermore, cytochrome *c* release can activate caspase-3, accelerating apoptosis and promoting myofibrillar degradation (Tang et al., 2024). Collectively, the predominant upregulation of DEPs in the Proteasome, Ribosome, and Oxidative phosphorylation pathways indicates that refrigerated storage triggers sustained cellular apoptosis, protein denaturation, oxidation, and degradation in tilapia fillets, ultimately contributing to quality deterioration.

### 3.6 Generation pathways of key volatile substances from protein degradation of Tilapia

Proteins serve as essential precursors for volatile substances, as the degradation of proteins and amino acids during refrigerated storage contributes to the formation of various volatile substances. However, the specific relationship between protein degradation and generation of volatile substances in refrigerated tilapia fillets remains poorly understood. To clarify this relationship, Pearson correlation analysis was performed between DEPs and key volatile substances. We identified DEPs that were significantly associated with key volatile substances and performed KEGG pathway enrichment analysis.

**Fig. 6:**
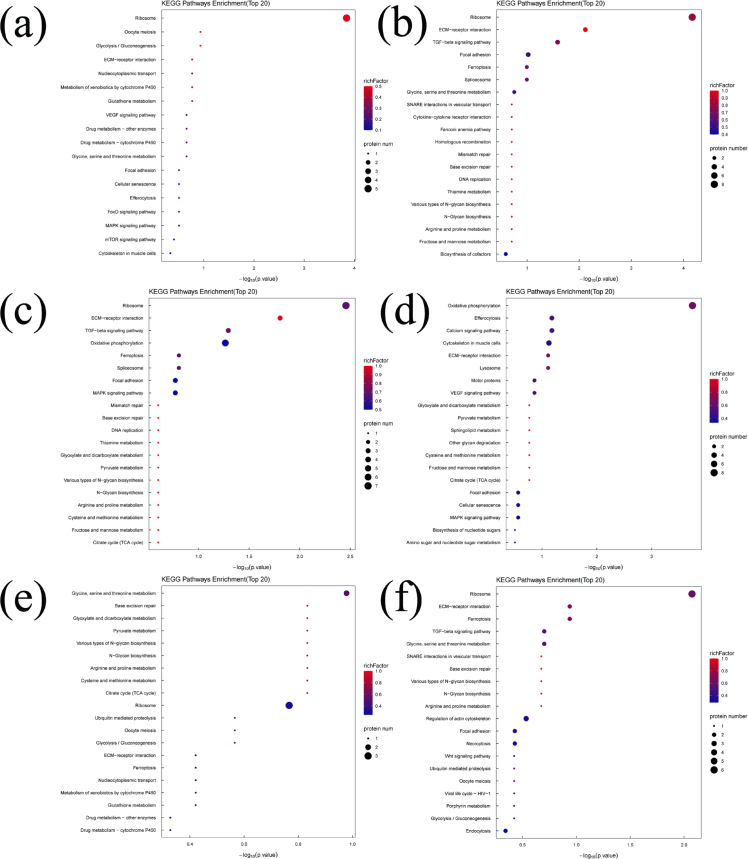

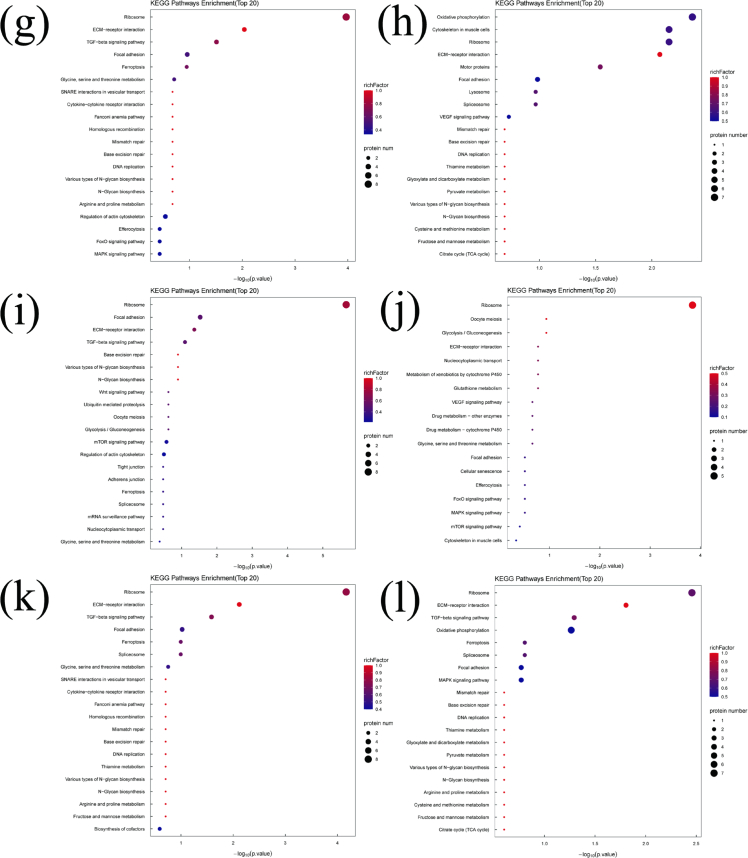

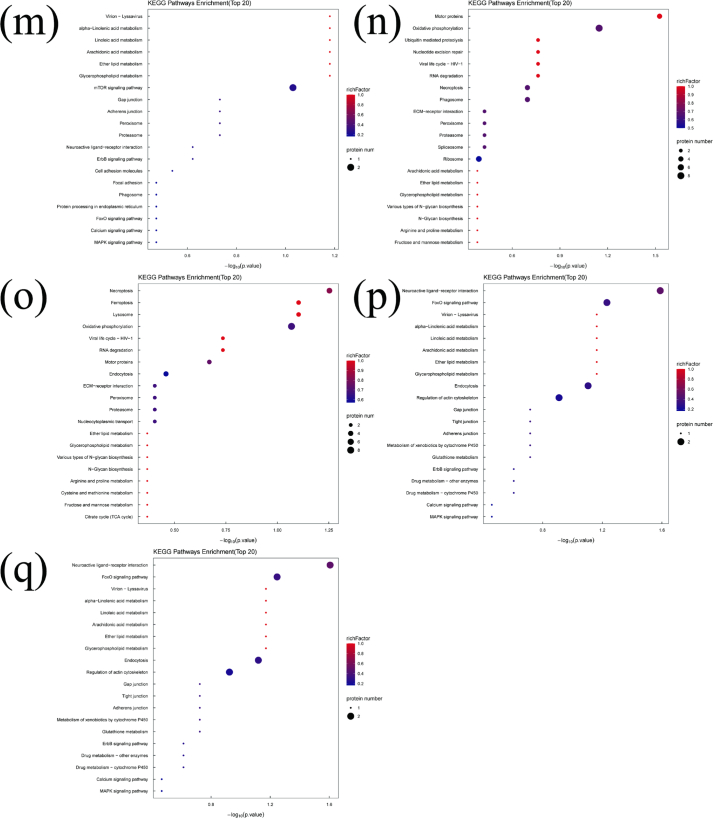
KEGG enrichment analysis of the DEPs related to key volatile substances during the refrigerated storage of tilapia fish. (a) Trimethylamine, (b) Pentanal, (c) Hexanal, (d) Heptanal, (e) Octanal, (f) Nonanal, (g) Decanal, (h) 1-Octen-3-ol, (i) (2E)-2-Decenal, (j) 6-Methyl-5-hepten-2-one, (k) Hexanol, (l) Heptanol, (m) Benzaldehyde, (n) (E)-2-Nonenal, (o) Octanol, (p) Butyric Acid, (q) Eugenol.

Bioinformatics analysis provides an effective approach for elucidating the relationship between DEPs and volatile substances formation during refrigerated storage. It also helps identify the metabolic pathways potentially involved in this process. KEGG pathway enrichment analysis was integrated with DEP–volatile substances correlation analysis. In this way, the metabolic processes potentially associated with the generation of key volatile substances were systematically characterized. The top 20 enriched KEGG pathways related to the DEPs of the 17 key volatile substances are shown in Fig. 6. The Ribosome pathway (onl03010) was significantly enriched (*P* < 0.05) in DEPs associated with trimethylamine, nonanal, 6-methyl-5-hepten-2-one, pentanal, decanal, hexanal, 1-octen-3-ol, (*2E*)-2-decenal, and hexanol (Table S3). The 40S ribosomal protein S8 (A0A669CYT8) was significantly upregulated (*P* < 0.05) in the trimethylamine-related pathway, and several ribosomal proteins (A0A669CYT8, A0A669E4L2, I3J3T8, I3JF29, and O13019) were significantly upregulated (*P* < 0.05) across pathways associated with nonanal, 6-methyl-5-hepten-2-one, pentanal, decanal, hexanal, 1-octen-3-ol, (*2E*)-2-decenal, and hexanol. Conversely, 60S ribosomal protein L26 (I3KME3) was significantly downregulated (*P* < 0.05) in the trimethylamine and 6-methyl-5-hepten-2-one pathways. Volatile substances formation is closely linked to oxidative degradation of proteins and amino acids. Upregulation of ribosomal proteins suggests accelerated ribosomal turnover, allowing extracellular peptidases to hydrolyze ribosomal proteins into peptides, dipeptides, tripeptides, and free amino acids—key substrates for volatile substances formation (Mora et al., 2015). Previous studies have also reported strong associations between certain fundamental ribosomal functions and changes in volatile profiles during storage (Liu, Liu, et al., 2020). The ECM-receptor interaction pathway (onl04512) was significantly enriched (*P* < 0.05) in DEPs associated with pentanal, decanal, hexanal, 1-octen-3-ol, and hexanol (Table S3). Fibronectin (I3J9G5) and dystroglycan 1 (I3KQF9) were significantly upregulated (*P* < 0.05). The TGF-β signaling pathway was enriched only in pentanal- and decanal-associated DEPs (Table S3), where serotransferrin (A0A0E3JRK1), RhoA-D (I3KRN4), and transforming growth factor-β (A0A0E3M0W2) were significantly upregulated (*P* < 0.05). The ECM-receptor interaction pathway regulates cell structure, adhesion, proliferation, differentiation, and migration, it also interacts with TGF-β signaling to modulate lipid metabolism (Ma et al., 2021). Upregulation of ECM components-such as laminin complexes reported in adipocyte differentiation (Aratani & Kitagawa, 1988)-may contribute to between lipid metabolism and volatile substances formation. Thus, activation of ECM-receptor interaction and TGF-β pathways may be associated with the generation of aldehydes and alcohols through lipid-derived metabolic routes. The oxidative phosphorylation pathway (onl00190) was significantly enriched (*P* < 0.05) in DEPs associated with heptanal, 1-octen-3-ol, and hexanol (Table S3). Upregulated DEPs included NADH dehydrogenase (A0A669CQS1, A0A669EMT2), cytochrome *c* oxidase (A0A669CXL8, I3KVG2), and ATP synthase (I3J116, I3K590) (*P* < 0.05). NADH dehydrogenase plays a crucial role in the redox reactions of the electron transport chain within the oxidative phosphorylation pathway, contributing to the production of ATP. Cytochrome c oxidase accepts electrons from cytochrome *c* molecules, transferring them to an oxygen molecule to convert oxygen into two water molecules. ATP synthase utilizes a rotary motor mechanism to convert adenosine diphosphate (ADP) into adenosine triphosphate (ATP), generating a high-energy compound (Zhang, Zhou, et al., 2019). Postmortem hypoxia in fish muscle shifts energy metabolism toward enhanced oxidative phosphorylation. Upregulation of these enzymes reflects increased ATP demand and mitochondrial activity. Thus, elevated oxidative phosphorylation appears to facilitate both the degradation of cellular components and volatile substances generation. Thirty-three KEGG pathways were associated with octanal (Table S3), among which glycine, serine, and threonine metabolism showed the most significant enrichment (*P* < 0.05). Guanidinoacetate *N*-methyltransferase (A0A669BYD3) was significantly upregulated (*P* < 0.05). Amino acids and their derivatives are fundamental precursors of volatile substances, with aldehydes and ketones often produced through amino acid transamination. Previous reports also confirm that alterations in amino acid metabolic pathways markedly affect volatile substances formation (Chu et al., 2023). Fifty KEGG pathways were associated with 1-octen-3-ol (Table S3). Based on significance analysis (*P* < 0.05), the cytoskeleton in muscle cells and motor protein pathways were uniquely enriched, aside from the Oxidative phosphorylation, Ribosome, and ECM-receptor interaction pathways. The motor protein pathway was also enriched among DEPs associated with hexanol and (*E*)-2-nonenal (Table S3). Within this pathway, tubulin beta chain (I3K020) was significantly upregulated (*P* < 0.05), while kinesin-like protein (I3IVI3) was significantly upregulated (*P* < 0.05) in hexanol- and (*E*)-2-nonenal-related pathways. The cytoskeleton-associated pathway was unique to 1-octen-3-ol (Table S3), with fibronectin (I3J9G5), myozenin-2 (I3JL52), and dystroglycan 1 (I3KQF9) showing significant upregulation (*P* < 0.05). Motor proteins generate mechanical force and drive intracellular movement. During fish fermentation, their degradation into peptides has been documented, and conformational changes in myosin may alter binding affinity to volatile substances (Wang et al., 2024). The cytoskeleton-composed of self-assembling polymers such as actin-is essential for maintaining cell morphology and biological functions. Following slaughter, caspase activation induces apoptosis in fish muscle, leading to progressive degradation of cytoskeletal and myofibrillar proteins, which may subsequently influence volatile substances formation (Tang et al., 2024). Thirty KEGG pathways were associated with (*2E*)-2-decenal (Table S3), and the Focal adhesion pathway was the only significantly enriched pathway (*P* < 0.05), aside from Ribosome and ECM-receptor interaction pathways. Fibronectin (I3J9G5) and RhoA-D (I3KRN4) were significantly upregulated (*P* < 0.05) in this pathway. Focal adhesions are dynamic protein complexes that anchor the cytoskeleton to the extracellular matrix (ECM). Previous studies on meat lipid metabolism demonstrate that genes involved in ECM-receptor interaction and Focal adhesion pathways are enriched among upregulated proteins, suggesting a role in regulating lipid-derived volatile substances formation (Liu, Liu, et al., 2020). Thus, upregulation of focal adhesion-related DEPs in this study may contribute to lipid metabolism processes influencing volatile substances generation. Twenty-three KEGG pathways were identified for benzaldehyde (Table S3), but only Glycerophospholipid metabolism was significantly enriched (*P* < 0.05). One protein was involved in this pathway. Phospholipids, rich in unsaturated fatty acids and reactive polar head groups, readily undergo oxidation in the presence of catalytic metals. They serve as key precursors of volatile substances and play an important role in flavor formation during meat storage and processing (Wu et al., 2024). Sixty-eight KEGG pathways were identified for octanol (Table S3), with the Necroptosis pathway being the only significantly enriched metabolic pathway (*P* < 0.05). Five proteins were involved, including high mobility group box 1 (A0A3G1Z143), charged multivesicular body protein 4b (A0A669E8E1), ferritin (I3J4F3), and caspase-8 (I3KF39), all of which were significantly upregulated (*P* < 0.05). Apoptosis and necroptosis are fundamental physiological events in postmortem muscle. During ATP generation for residual metabolic activity, protein degradation accelerates, providing substrates for volatile substances formation. Thus, the upregulation of necroptosis-related DEPs may be associated with enzyme-mediated protein breakdown, ultimately contributing to volatile substances production (Yan et al., 2023). For butyric acid, eugenol, and heptanol, only one significantly enriched pathway was identified: Neuroactive ligand-receptor interaction, with 24, 24, and 19 pathways associated with each volatile substance, respectively (Table S3). Two identical DEPs were present in this pathway, and Complement C5 (I3IW28) was significantly downregulated (*P* < 0.05). Studies have shown that metabolic biomarkers can regulate lipid metabolism through the Neuroactive ligand-receptor interaction pathway, thereby influencing the formation of lipid-derived volatile substances (Xie et al., 2024). The downregulation of complement proteins observed here suggests potential regulatory effects on fatty acid metabolism, which may impact the production of these volatile substances.

### 3.7 Pathway map for potential generation of key volatile substances

**Fig. 7:**
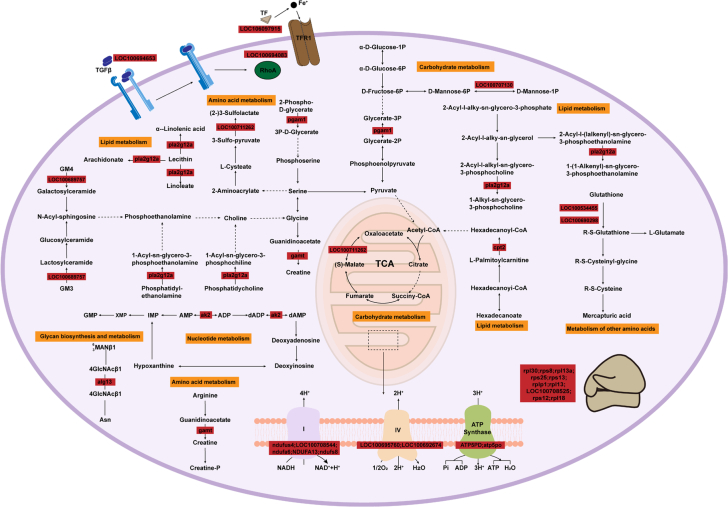
Pathway map for potential generation of key volatile substances.

In the KEGG enrichment analysis, metabolic pathways such as amino acid metabolism, lipid metabolism, nucleotide metabolism, and carbohydrate metabolism were found to be closely associated with the formation of key volatile substances. To further clarify the mechanisms underlying the generation of these volatile substances during refrigerated storage, a potential metabolic pathway map was constructed (Fig. 7). Lipid metabolism plays a major role in volatile substances generation (Liu, Crane, et al., 2024). Lecithin is hydrolyzed by secretory phospholipase A2 (pla2g12a) to produce arachidonic acid, linoleic acid, and α-linolenic acid. These unsaturated fatty acids undergo β-oxidation and participate in arachidonic acid, linoleic acid, and α-linolenic acid metabolic pathways, ultimately producing key aldehydes and alcohols such as hexanal, octanal, nonanal, decanal, and 1-octen-3-ol (Liu, Wei, et al., 2024). Secretory phospholipase A2 also contributes to ether lipid and glycerophospholipid metabolism, further supporting the formation of lipid-derived volatile substances. In fatty acid degradation, hexadecanoate undergoes oxidative breakdown via carnitine O-palmitoyltransferase 2 (cpt2), providing energy for metabolic processes. In purine metabolism, ATP degradation generates ADP, which, through the catalytic action of adenylate kinase (ak2), can be converted into one ATP and one AMP from two ADP molecules. AMP is subsequently deaminated to inosine monophosphate (IMP). Both ADP and IMP are recognized contributors to flavor development (Liu, Crane, et al., 2024), suggesting that nucleotide catabolism plays a supporting role in volatile substances precursor formation. Amino acids, one of the major components of non-protein nitrogen, serve as essential precursors for volatile substances formation (Chu et al., 2023). Glutathione, an important antioxidant in fish muscle, is degraded during refrigerated storage under oxidative stress. This process, catalyzed by glutathione S-transferases (LOC100534455, LOC100690298), liberates amino acids that support volatile substances production (Liu, Deng, et al., 2024). 2-Phospho-D-glycerate can be converted into serine, glycine, and threonine by 2,3-bisphosphoglycerate-dependent phosphoglycerate mutase (pgam1), and these amino acids influence both meat quality and volatile substances formation (Gai et al., 2023). Serine is further converted into pyruvate by guanidinoacetate *N*-methyltransferase (gamt) and malate dehydrogenase (LOC100711262), which then enters the citrate cycle to supply energy. Arginine may also contribute to the diversity of volatile substances, particularly enhancing the production of ketones (Dou et al., 2023). Pyruvate, a central metabolite in pyruvate metabolism, amino sugar and nucleotide sugar metabolism, and glycolysis/gluconeogenesis, is produced through the action of malate dehydrogenase (LOC100711262), phosphomannomutase (LOC100707130), and pgam1. Once generated, pyruvate enters the citrate cycle to support energy production during refrigerated storage. Meanwhile, amino sugar and nucleotide sugar metabolism and glycolysis/gluconeogenesis generate organic acids, which serve as important precursors for the formation of volatile substances.

### 3.8 Limitations and future perspectives

Although the present study provides valuable insights into the relationship between proteomic changes and volatile substances formation during refrigerated storage of tilapia fillets, several limitations should be acknowledged. Lipid oxidation, microbial growth, and free amino acid changes were not directly measured. This limits further verification of the specific contributions of oxidized lipids to aldehyde, ketone, and alcohol, of microbial metabolism to spoilage-related odor formation, and of free amino acids to volatile substances generation through deamination, decarboxylation, and related reactions. Therefore, some of the proposed mechanisms remain inferential and are mainly based on proteomic analysis combined with volatile profiling, correlation analysis, and pathway enrichment analysis. Future studies integrating these indicators with proteomics and flavoromics would provide stronger evidence for the biochemical and microbial mechanisms underlying quality deterioration and volatile substances formation during refrigerated storage.

## 4 Conclusion

In this study, HS-SPME-GC–MS combined with Astral DIA proteomics was used to investigate changes in volatile substances and DEPs in tilapia fillets stored at 4 °C for 7 days. Pearson correlation analysis and KEGG enrichment analysis were further employed to elucidate the metabolic pathways linking protein degradation to the formation of key volatile substances. Seventeen key volatile substances (OAV > 1) were identified during refrigerated storage, and their levels progressively increased, contributing fishy, mushroom-like, and earthy odors characteristic of quality deterioration. Five key volatile substances were identified by the PLS-DA model and may serve as potential markers for sample classification. Among these volatile substances, trimethylamine can serve as one of the indicators for freshness monitoring and may be used to evaluate the freshness of aquatic products through color changes in membrane-based indicators. Aldehydes, in contrast, can be regarded as representative markers associated with lipid oxidation and flavor deterioration (Huang et al., 2023; Phetsang et al., 2021). Astral DIA Proteomics revealed that DEPs were mainly enriched in the Proteasome, Ribosome, and Oxidative phosphorylation pathways. Continuous apoptosis and oxidative degradation mediated by Proteasome, Ribosome, ATP synthase, Cytochrome c oxidase, and NADH dehydrogenase contributed to cellular damage and loss of muscle quality during storage. Among the 17 key volatile substances, pathways such as Ribosome, ECM-receptor interaction, Oxidative phosphorylation, and Neuroactive ligand-receptor interaction were closely associated with the formation of trimethylamine, nonanal, 6-methyl-5-hepten-2-one, pentanal, decanal, hexanal, heptanal, 1-octen-3-ol, (*2E*)-2-decenal, hexanol, butyric acid, eugenol, and heptanol. These associations may involve protein degradation, energy metabolism, and lipid metabolic regulation. Furthermore, amino acid, lipid, nucleotide, and carbohydrate metabolism directly facilitated the formation of key volatile substances through enzymatic actions involving glutathione S-transferase, secretory phospholipase A2, adenylate kinase, and malate dehydrogenase. Based on these results, a potential metabolic pathway map was constructed to illustrate the biochemical routes leading to volatile substances formation. Overall, this study demonstrates the central role of protein degradation and associated metabolic pathways in driving flavor deterioration in tilapia fillets during refrigerated storage. By integrating Astral DIA proteomics with volatile profiling, this work provides molecular-level mechanistic insights that may guide strategies to control protein degradation and improve the flavor quality of fish products under refrigerated conditions.

## CRediT authorship contribution statement

**Zhenyang Liu:** Writing – review & editing, Writing – original draft, Methodology, Investigation, Formal analysis. **Naiyong Xiao:** Resources, Formal analysis, Data curation. **Lei Qin:** Resources, Formal analysis, Data curation. **Zefu Wang:** Resources, Formal analysis, Data curation. **Qinxiu Sun:** Resources, Formal analysis, Data curation. **Hui Cao:** Writing – review & editing, Supervision. **Shucheng Liu:** Writing – review & editing, Project administration, Funding acquisition, Conceptualization.

## Ethics statement

All animal research was conducted in accordance with current guidelines of the Experimental Animal Ethics Committee of Guangdong Ocean University (Approval No. GDOU-LAE-2023-043).

## Declaration of competing interest

The authors declare that they have no known competing financial interests or personal relationships that could have appeared to influence the work reported in this paper.

## Data Availability

Data will be made available on request.
