# Supplementary material for: Insights into protein degradation-related volatile substances changes in tilapia fillets (Oreochromis niloticus) during storage at 4 °C based on astral DIA proteomics and flavoromics
Source: Food Chem X. 2026 Apr 21;36:103895. doi: 10.1016/j.fochx.2026.103895 (PMC13129469; doi:10.1016/j.fochx.2026.103895)
Supplement: Supplementary file 1 — Supplementary material 1 [file mmc1.docx]

**Supplementary materials**

**Fig. S1** Subloc classification (a-d) and domain classification (e-h) of DEPs in tilapia fillets during cold storage. (a)(e) d1/d0, (b)(f) d3/d0, (c)(g) d5/d0, (d)(h) d7/d0

**Fig. S2** KEGG pathway mapping of the DEPs in tilapia fillets during cold storage. (a) d1/d0, Proteasome; (b) d3/d0, Ribosome; (c) d5/d0, Oxidative phosphorylation; (d) d7/d0, Oxidative phosphorylation

**Table S1** Concentrations (mg/kg) of the volatile substances detected in tilapia fillets with different storage times

**Table S2** Odour activity values (OAV≥1) for volatile substances of tilapia fillets with different storage times


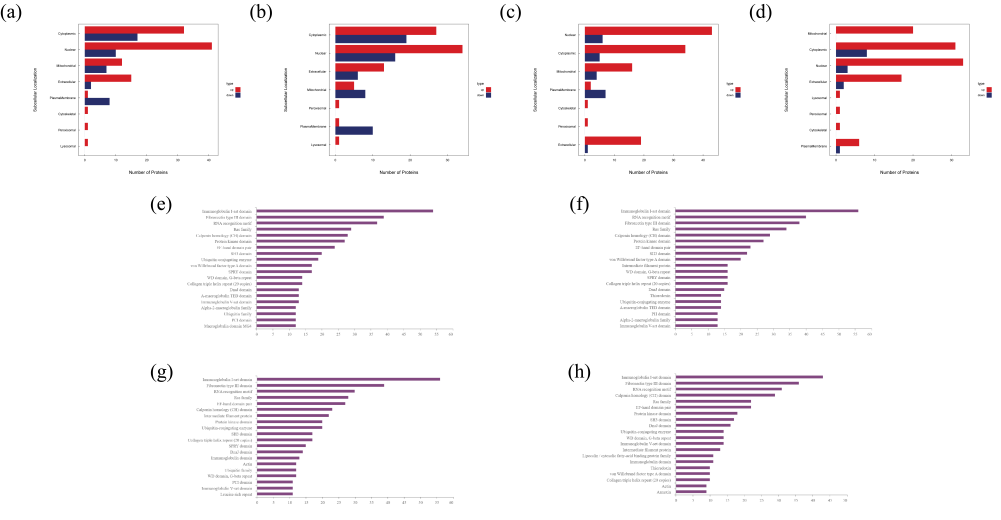


**Fig. S1** Subloc classification (a-d) and domain classification (e-h) of DEPs in tilapia fillets during cold storage. (a)(e) d1/d0, (b)(f) d3/d0, (c)(g) d5/d0, (d)(h) d7/d0


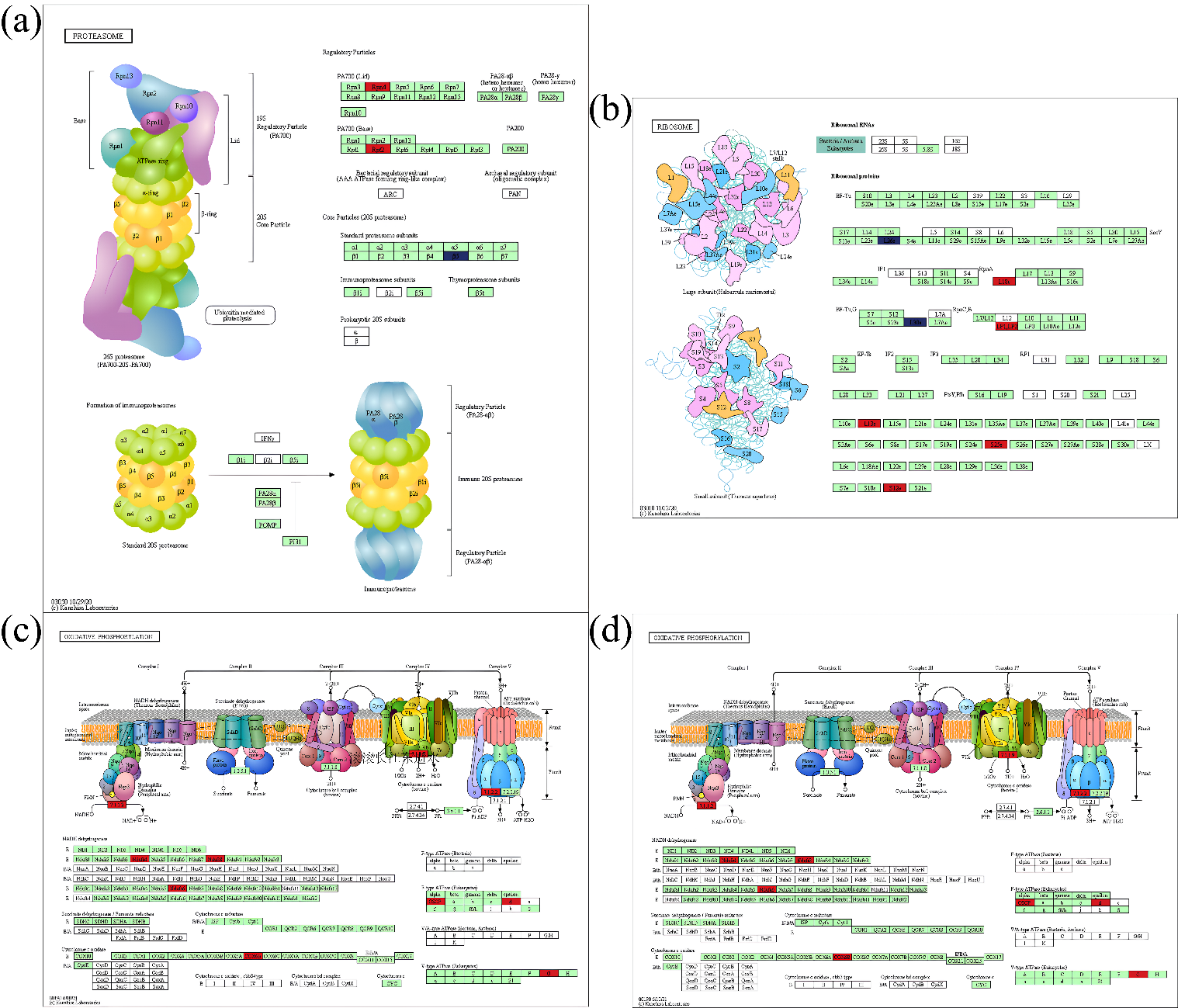


**Fig. S2** KEGG pathway mapping of the DEPs in tilapia fillets during cold storage. (a) d1/d0, Proteasome; (b) d3/d0, Ribosome; (c) d5/d0, Oxidative phosphorylation; (d) d7/d0, Oxidative phosphorylation

**Table S1** Concentrations (mg/kg) of the volatile substances detected in tilapia fillets with different storage times

| CAS | Compounds name | Identification | 0d | 1d | 3d | 5d | 7d |
| --- | --- | --- | --- | --- | --- | --- | --- |
|  | Alcohols (7) |  |  |  |  |  |  |
| 616-25-1 | 1-Penten-3-ol | MS, RI | 0.69±0.40ab | 0.79±0.46a | 0.69±0.35ab | 0.64±0.37b | 0.64±0.37b |
| 71-41-0 | 1-Pentanol | MS, RI | 1.36±0.42ab | 1.39±0.81ab | 1.53±0.89a | 0.99±0.16b | 1.74±1.01a |
| 111-27-3 | 1-Hexanol | MS, RI | 0.67±0.27a | 0.83±0.13a | 0.92±0.21a | 0.57±0.07a | 0.94±0.45a |
| 3391-86-4 | 1-Octen-3-ol | MS, RI | 1.49±0.25c | 2.44±0.39b | 2.72±0.38ab | 1.65±0.03bc | 3.43±0.65a |
| 111-70-6 | 1-Heptanol | MS, RI | 1.88±0.14ab | 1.99±0.12a | 1.38±0.37abc | 1.22±0.35bc | 1.15±0.45c |
| 104-76-7 | 2-Ethylhexanol | MS, RI | 3.59±0.42a | 3.21±1.62ab | 3.17±1.83ab | 3.57±0.31a | 2.83±0.17b |
| 111-87-5 | 1-Octanol | MS, RI | 0.60±0.09b | 1.06±0.23ab | 0.62±0.12ab | 0.64±0.04ab | 1.20±0.48a |
|  | Ketones (4) |  |  |  |  |  |  |
| 513-86-0 | 3-hydroxy-2-oxobutane | MS, RI | 0.2±0.01b | 0.42±0.09ab | 0.63±0.33a | 0.49±0.02ab | 0.48±0.24ab |
| 565-80-0 | 2,4-Dimethyl-3-pentanone | MS, RI | 1.09±0.54a | 1.67±0.85a | 0.97±0.74a | 0.88±0.18a | 1.91±1.70a |
| 110-93-0 | 6-Methyl-5-hepten-2-one | MS, RI | 0.55±0.08a | 0.67±0.13a | 0.62±0.21a | 0.87±0.16a | 0.9±0.22a |
| 629-66-3 | 2-Nonadecanone | MS, RI | 0.67±0.35a | 0.83±0.45a | 0.79±0.41a | 0.38±0.22b | 0.29±0.04b |
|  | Aldehydes (11) |  |  |  |  |  |  |
| 110-62-3 | Pentanal | MS, RI | 6.75±1.86b | 7.40±0.79b | 8.10±0.61b | 8.61±0.27ab | 10.8±5.36a |
| 66-25-1 | Hexanal | MS, RI | 10.68±0.57b | 16.64±0.66b | 16.90±0.61b | 19.11±10.78ab | 30.72±6.5a |
| 111-71-7 | Heptaldehyde | MS, RI | 2.36±0.36a | 2.52±0.51a | 2.65±0.65a | 1.76±0.26a | 2.95±0.43a |
| 124-13-0 | Octanal | MS, RI | 1.17±0.27b | 1.56±0.04ab | 0.86±0.23b | 1.61±0.03ab | 2.33±0.73a |
| 124-19-6 | 1-Nonanal | MS, RI | 4.98±0.49bc | 6.10±0.99abc | 4.54±1.58c | 6.84±0.43ab | 7.97±3.25a |
| 112-31-2 | Decanal | MS, RI | 1.80±0.14b | 1.95±0.19b | 1.95±0.43b | 2.40±0.08ab | 2.73±0.36a |
| 100-52-7 | Benzaldehyde | MS, RI | 1.22±0.07a | 1.28±0.08a | 1.26±0.28a | 1.37±0.24a | 0.95±0.18a |
| 18829-56-6 | trans-2-Nonenal | MS, RI | 0.35±0.10b | 0.69±0.03a | 0.73±0.06a | 0.75±0.06a | 0.43±0.13b |
| 3913-81-3 | trans-2-Decenal | MS, RI | 0.82±0.05a | 0.85±0.11a | 0.89±0.05a | 0.9±0.21a | 1.10±0.17a |
| 1337-83-3 | trans-2-Undecenal | MS, RI | 0.26±0.07a | 0.41±0.19a | 0.32±0.11a | 0.33±0.19a | 0.37±0.28a |
| 124-25-4 | Tetradecanal | MS, RI | 1.20±0.34c | 0.71±0.08ab | 0.59±0.06bc | 0.57±0.17a | 0.42±0.04c |
|  | Acids (8) |  |  |  |  |  |  |
| 64-19-7 | Acetic acid | MS, RI | 4.25±0.28ab | 4.44±0.48a | 3.80±0.87abc | 2.94±0.34bc | 2.75±0.7c |
| 1979/9/4 | Propionic acid | MS, RI | 0.79±0.12a | 0.64±0.19ab | 0.48±0.24b |  |  |
| 107-92-6 | Butyric Acid | MS, RI | 8.06±1.12a | 7.39±0.22ab | 6.96±1.07ab | 5.76±1.22ab | 5.21±1.93b |
| 112-05-0 | Nonanoic acid | MS, RI | 0.63±0.36a | 0.55±0.31ab | 0.53±0.27ab | 0.50±0.24bc | 0.42±0.28c |
| 142-62-1 | Hexanoic acid | MS, RI | 0.74±0.24b | 0.77±0.41b | 0.96±0.10ab | 0.98±0.59ab | 1.05±0.60a |
| 111-14-8 | Heptanoic acid | MS, RI | 0.60±0.31a | 0.51±0.28ab | 0.47±0.23ab | 0.38±0.15bc | 0.29±0.11c |
| 124-07-2 | Octanoic acid | MS, RI | 1.19±0.66b | 2.97±1.68a | 1.34±0.70b | 0.37±0.21c | 0.25±0.13c |
| 334-48-5 | Decanoic acid | MS, RI | 0.52±0.31c | 0.65±0.48b | 1.48±0.74a | 0.51±0.30c | 0.2±0.11d |
|  | Esters (2) |  |  |  |  |  |  |
| 141-32-2 | Butyl acrylate | MS, RI | 0.45±0.23c | 0.48±0.25c | 0.81±0.67b | 1.29±0.08a | 1.33±0.77a |
| 109-21-7 | Butyl butyrate | MS, RI | 0.52±0.23b | 0.52±0.26b | 0.56±0.38b | 0.93±0.22a | 1±0.58a |
|  | Nitrogen compounds (1) |  |  |  |  |  |  |
| 75-50-3 | Trimethylamine | MS, RI |  |  |  | 0.33±0.03b | 11.44±3.18a |
|  | Sulfur compounds (1) |  |  |  |  |  |  |
| 686-07-7 | methyl diethyldithiocarbamate | MS, RI | 0.56±0.29b | 1.44±0.76a | 1.48±0.78a | 1.59±0.92a | 1.74±1.29a |
|  | Hydrocarbons (9) |  |  |  |  |  |  |
| 138-86-3 | DL-Limonene | MS, RI | 0.60±0.09ab | 0.68±0.12ab | 0.57±0.31b | 0.75±0.10ab | 0.85±0.16a |
| 112-40-3 | Dodecane | MS, RI | 0.92±0.15a | 1.27±0.46a | 1.28±0.52a | 1.52±0.35a | 1.45±0.77a |
| 31295-56-4 | 2,6,11-trimethylDodecane | MS, RI | 0.99±0.12ab | 1.03±0.34a | 0.86±0.50b | 0.85±0.10b | 0.84±0.04b |
| 629-50-5 | n-Tridecane | MS, RI | 0.72±0.13a | 0.84±0.13b | 0.79±0.35a | 0.89±0.25b | 0.89±0.48b |
| 544-76-3 | n-Hexadecane | MS, RI | 0.44±0.03a | 0.51±0.08a | 0.56±0.12a | 0.62±0.06a | 0.62±0.22a |
| 475-20-7 | D-longifolene | MS, RI | 0.68±0.14b | 0.71±0.33b | 0.77±0.49ab | 0.90±0.52a | 0.88±0.51a |
| 275-51-4 | Azulene | MS, RI | 0.58±0.14ab | 0.62±0.33ab | 0.70±0.36ab | 0.52±0.30b | 0.74±0.67a |
| 108-95-2 | Phenol | MS, RI | 0.49±0.25ab | 0.54±0.27ab | 0.48±0.29b | 0.55±0.32ab | 0.60±0.35a |
| 97-53-0 | Eugenol | MS, RI | 1.47±0.13a | 1.40±0.22a | 1.28±0.23ab | 1.13±0.01a | 1.01±0.31b |

Data are mean ± standard deviation (n = 3). Different letters within a row indicate significant difference (*P* < 0.05).

**Table S2** Odour activity values (OAV≥1) for volatile substances of tilapia fillets with different storage times

| Compounds name | 0 d | 1 d | 3 d | 5 d | 7 d |
| --- | --- | --- | --- | --- | --- |
| Trimethylamine | 0 | 0 | 0 | 14.56 | 497.41 |
| Pentanal | 844.23 | 924.92 | 1012.01 | 1076.54 | 1350.49 |
| Hexanal | 1423.45 | 2218.07 | 2253.18 | 2548.04 | 4095.41 |
| Heptanal | 236.02 | 251.71 | 265.48 | 176.36 | 294.98 |
| Octanal | 1664.53 | 2249.01 | 1228.07 | 2302.68 | 3324.49 |
| 6-Methyl-5-hepten-2-one | 5.49 | 6.72 | 6.24 | 8.71 | 8.99 |
| Hexanol | 0.95 | 1.19 | 1.32 | 0.82 | 1.34 |
| Nonanal | 332.02 | 406.52 | 302.49 | 455.83 | 531.54 |
| 1-Octen-3-ol | 213.46 | 348.36 | 388.72 | 235.34 | 490.55 |
| Heptanol | 9.4 | 9.96 | 6.91 | 6.09 | 5.74 |
| Decanal | 360.68 | 390.36 | 390.29 | 479.35 | 545.43 |
| Benzaldehyde | 4.07 | 4.26 | 4.21 | 4.56 | 3.17 |
| (*E*)-2-Nonenal | 5384.62 | 10615.38 | 11230.77 | 11538.46 | 6615.38 |
| Octanol | 11.13 | 19.55 | 11.41 | 11.87 | 16.57 |
| Butyric Acid | 2.95 | 2.71 | 2.55 | 2.11 | 1.91 |
| (*2E*)-2-Decenal | 5.47 | 5.68 | 5.95 | 6.01 | 7.36 |
| Eugenol | 1474.64 | 1398.65 | 1279.86 | 1132.7 | 1007.73 |
